# Reference checking for systematic reviews using Endnote

**DOI:** 10.5195/jmla.2018.489

**Published:** 2018-10-01

**Authors:** Wichor M. Bramer

**Affiliations:** Biomedical Information Specialist, Medical Library, Erasmus MC–Erasmus University Medical Centre, Rotterdam, The Netherlands

## Abstract

In searches for systematic reviews, it is recommended that authors review references from the reference lists of retrieved relevant reviews for possible additional, relevant references. This process can be time consuming, since there often is overlap between the reference lists and the lists contain references that were already retrieved in the initial searches. The author proposes a method in which EndNote is used in combination with the Scopus or Web of Science databases to semi-automatically download these references into an existing EndNote library.

Most guidelines for searches for systematic reviews recommend that, next to searching traditional databases, alternative methods are used to find relevant articles. One alternative method is to review the reference lists of relevant reviews and already included citations to find articles that were not yet retrieved [1–3]. In the experience of Chapman et al., the new content can add 5%–10% to the citations found [4]. These extra references do add to the burden for the reviewer, especially since items on the reference lists often overlap and might have already been found by the database searches. Nevertheless, other studies have found that searching reference lists can add great value to database searches and should, therefore, not be omitted [5].

The author proposes a method that allows deduplication between these reference lists both internally and against the existing content that was downloaded from bibliographic database searches. Chapman et al. investigated the validity of a similar method, compared to manual screening of the reference lists [4]. They found that the method saved the time to screen the reference lists by 62.5%. However, they did not provide enough detail on how to execute each step, and the time needed for their method seems substantial. This article describes in detail the steps that can be used to efficiently perform my method. The method described by Chapman et al. has been improved by automating the Scopus search with a specially designed output style from EndNote that allows automatic searching of articles from an existing EndNote library in Scopus, allowing faster downloading of the reference lists than reported in Chapman et al.’s article [4].

## AUTOMATIC DOWNLOADING OF REFERENCE LISTS OF INCLUDED REFERENCES

Several databases allow users to download cited or citing references of a list of articles. Two of such databases are Scopus by Elsevier [6] and ISI Web of Science by Clarivate Analytics [7]. They both need a subscription to be accessed. If a researcher or librarian has access to one of these databases, custom-made export formats from EndNote can create strategies for the selected references in those databases, leading the searcher to extra, relevant articles.

For this purpose, the functionality of Scopus is superior to that of Web of Science, because Scopus can export the cited and citing references of a list of articles simultaneously. Web of Science, on the other hand, is only able to deliver those lists per individual reference, making the process much more cumbersome and time consuming. Using Web of Science, a researcher can export all reference lists individually, and therefore, the reference lists have to be deduplicated internally before being compared to the existing list of references retrieved from the bibliographic database. Results exported from Scopus are already internally deduplicated, so an article cited by more than one reference is present only once.

Also, I have found that Scopus was able to retrieve more articles via this search option than Web of Science. Nevertheless, I describe the methods in both Scopus and Web of Science.

## THE METHOD

### Step 1: Change the EndNote settings as preparation

First, the EndNote settings have to be prepared for this method. Special Output Styles have to be installed. Also, a column showing page numbers of references in EndNote should be made visible. This allows the selection of papers that have page numbers as they will be searched differently than those that do not.

1. Go to bit.ly/emcendnotes.
2. Open the zip file.
3. Open the file _scopus search.ens.
4. In EndNote, select File > Save as.
5. Remove Copy from the file name and Click [Save].
6. Repeat steps 3–5 for the files _scopus pmid.ens,_scopus title.ens,_wos search.ens, _wos pmid.ens, and _wos title.ens (depending on the platform you choose to use).
7. Go to Edit > Preferences > Display Fields.
8. For one of the columns (preferably, one of the higher numbered columns), select Pages from the drop-down menu and click OK.
9. Change the deduplication settings: Go to: Edit > Preferences > Duplicates. Select Year, Title and click OK.

### Step 2: Prepare an EndNote library containing the relevant references

Create a new EndNote library that contains only those articles for which you want to perform the reference check: included references and relevant reviews. In that EndNote library, create a group named “resolved” and a group named “unmatched” (right click on My Groups > Create Group).

### Step 3: Use EndNote to create a search for the relevant references

In the following steps, EndNote creates a search strategy that will search for the relevant landmark references in the database of choice. Generally, the results from systematic review searches and, therefore, the EndNote representation of included references originate from many different databases and interfaces. All of these databases store their data in different ways. It can, therefore, be hard to find these references directly in the databases. This means the search has to be done in multiple steps.

1. From the drop-down menu in the top left corner of EndNote (Style selection), select the style _scopus pmid when using Scopus. (If you plan to use Web of Science, select the style _wos pmid.)
2. Select one random reference in All References and press Ctrl-A to select all references. Press Ctrl-K to copy the search to your clipboard.
3. In Scopus: Go to Advanced, click on Enter query string, press Ctrl-V to paste your search strategy, and click Search.
4. In Web of Science: Click on Advanced Search in the menu. Paste the search strategy in the search field. Remove the last occurrence of the Boolean operator “OR” and click Search.

### Step 4: Mark the references that have been resolved

The next steps are meant to distinguish the references that you have already found in the database from the ones that you have not yet found. Import the references that have been detected in Scopus or Web of Science, and deduplicate these with the remaining articles for which references should be checked. After deduplication, the references that have been resolved will be placed in a special group.

1. Download the references of the articles that were retrieved:
  In Scopus: Check the box before All in the top left corner of the results. Open the drop-down menu for Export, and select “RIS format.” Choose “Citation information only” from the drop-down menu and click export. Open the file. The contents will be imported in EndNote. In Web of Science: In the drop-down menu at the top of the results, select Save to EndNote Desktop. In the next screen, type in the text boxes after Records 1 to the total number of hits (if that is more than 500, limit to 500, and come back later for 501 and further). Open the file. The contents will be imported in EndNote.
2. Go to All References, and click on one random reference.
3. In the menu, select References > Find Duplicates.
4. In the next window, the Find Duplicates selection window, click on [Cancel].
5. Press delete to remove all highlighted duplicates.
6. Go to the Duplicate References group and press Ctrl-A. Drag these references into the “resolved” group.
7. Go to the Imported References, select one random reference, and press Ctrl-A.
8. Go to Unfiled and click on the heading Title to sort by Title.
9. Scan the list for references marked in blue, and hold Ctrl while clicking on similar references directly above or below the marked reference. If a marked reference does not have a similar reference directly above or below it, unmark it with Ctrl-Click.
10. After all duplicates have been selected, drag the marked references to resolved.
11. Go to resolved, select one random reference, and press Ctrl-A.
12. Go to Imported References, click on the scroll bar (if no scroll bar is shown, Ctrl-Click on one of the references, and Ctrl-Click again) and press Delete.
13. If references remain in the Imported References, these are unmatched references that were found in Web of Science or Scopus but were not among the original relevant references. If that is the case, drag the remaining references from imported to the unmatched group.

### Step 5: Repeat steps 3 and 4 for other searches

Now, steps 3 and 4 are repeated for other searches. The “Unfiled” group contains references that are not yet dragged to the “resolved” group. Follow the steps above with the following directions. To make sure that the more sensitive search for page numbers and author names does not identify false references, you need to sort the library in a way so that you can select only those references with more than two authors and page numbers higher than fifty.

1. Select the style:
  In Scopus: _scopus pages
  In Web of Science: _wos pages
2. Go to Unfiled.
3. In the menu, go to Tools > Sort Library.
4. Select Sort First by: # of Authors, and Then by: Pages and click [OK].
5. Scroll down and click on the first reference with 2 authors and a page number higher than 50. Now, press Shift-End to select all references from there to the bottom of the list.
6. Click on the Column heading Pages. Hold Ctrl while clicking on the first reference without a page number. Next, hold Ctrl-Shift while clicking the first reference with a page number higher than 50.
7. Press Ctrl-K.
8. In Scopus: Go to Advanced, click on Enter query string, and press Ctrl-V to paste your search strategy and click Search.
  In Web of Science: Select Advanced Search from the drop-down menu. Paste the search strategy in the search field. Remove the last occurrence of the Boolean operator “OR” and click Search.
9. Follow Step 4 as described above.
10. Select the style:
  In Scopus: _scopus title
  In Web of Science: _wos title
11. Go to Unfiled, select one random reference and press Ctrl-A.
12. Press Ctrl-K
13. In Scopus: Go to Advanced, click on Enter query string, and press Ctrl-V to paste your search strategy and click Search.
  In Web of Science: Select Advanced Search from the drop-down menu. Paste the search strategy in the search field. Remove the last occurrence of the Boolean operator “OR” and click Search.
14. Follow Step 4 as described above.

If the error “The search contains an incorrect fieldname” appears, this is probably because some of the titles contain *parentheses* or *equal to* signs. Solve that with the following steps:

- Go to Edit > Find and Replace.
- Select in the field: Title, with Find type “(”, deselect Match Words, and click [Change].
- Repeat steps a and b for “)” and “=.”

Not all references might have been found with these methods. If references remain in Unfiled, they have not been found in the databases, which means that the reference lists of these articles have to be scanned manually.

### Step 6: Remove false results

Some references might have been accidentally retrieved by the searches. They can be found in the unmatched group. If that is the case, these articles should be removed from the search results before the reference lists are exported.

1. Go to the unmatched group and click on the Author column heading.
2. Press Ctrl-A, followed by Ctrl-K.
3. In Scopus or Web of Science, go to Advanced Search and paste and execute the search strategy.

### Step 6: Download the cited and citing references

Now that most relevant articles have been found in the databases, the references can be downloaded into the original EndNote file containing the search results of the original searches.

#### In Scopus

1. Go to advanced search and look at the search history.
2. Combine the searches from the history that contain references; usually, these are the only searches in the history: #1 OR #2 OR #3.
3. If unmatched references were found, subtract that search strategy from the results: #1 OR #2 OR #3 AND NOT #4.
4. Click on the arrow right next to the top check box, and check the box before check all.
5. Click More > View References to view the Cited references.
6. If the number of references is higher than 2,000, use filters in the column Refine Results to create 2 or more separate result sets that contain fewer than 2,000 hits. For instance, select a set of publication years. First, click on limit to and execute the steps below, and later, select the same years and select Exclude.
7. In that overview again, click on the arrow right next to the top check box, and check the box before check all.
8. Click Export, select RIS Format, and select Citations and abstract information.
9. Open the resulting file Scopus.ris to import the references in Endnote (import them in the file where all included and excluded are).
10. Go back to Scopus and click on View Cited By to see an overview of citing references.
11. Repeat actions 6–-9 to import citing references.
12. Now, deduplicate the file to remove those articles that have already been reviewed from the new references using the method desribed in an earlier article [8].

#### In Web of Science

1. If the search results are satisfying, click on the number of hits in the search history in the table at the bottom of the page
2. Click on the first result.
3. On the left side of the screen, two links are visible: X times cited and Y Cited References. Clicking on the first link results in the citing references (thus, newer related articles). Clicking on the second provides the complete reference list of this article; therefore, finding older related citations. Both lists have to be exported separately for each article and imported into the existing EndNote library containing the references retrieved by the database searches.
4. If this is done for the first record, continue with the second, and so on.
5. Now, deduplicate the file to remove those articles that have already been reviewed from the new references using the method described in an earlier article [8].

## DISCUSSION

Advantages of the method are that screening of the references is easier as the downloaded citations also contain the abstracts of the articles, whereas when the references are only reviewed in the reference lists of the citing articles, only bibliographic information is shown, and the reviewer has to decide based on the title whether a citation might be relevant. Using the method described in the article by Bramer et al. in this issue of the *Journal of the Medical Library Association* [9], the screening process can be performed as is normally done for references.

Another advantage of the method is that unnecessary screening of duplicate references is avoided. References that are cited by more than one of the core articles are downloaded only once from the databases, and additionally the complete list of references is deduplicated with the references that had already been retrieved by the database searches.

Disadvantages are that not every article is indexed in Scopus and that, for some indexed articles, the references lists cannot be downloaded. This means that for some core articles, the traditional method of reference checking should still be followed. However, this is not a true disadvantage of the method, as in the traditional methods, this should be done as well, but for all references. Also, I noted that some of the references found in Scopus do not have all data available for download. Sometimes, titles are missing. This will mostly occur with rather old and general, non-journal article references, so this probably will not result in missed relevant references.
